# Machine Learning Models to Establish the Risk of Being a Carrier of Multidrug-Resistant Bacteria upon Admission to the ICU

**DOI:** 10.3390/antibiotics14090889

**Published:** 2025-09-03

**Authors:** Sulamita Carvalho-Brugger, Mar Miralbés Torner, Gabriel Jiménez Jiménez, Montserrat Vallverdú Vidal, Begoña Balsera Garrido, Xavier Nuvials Casals, Mercedes Palomar Martínez, Javier Trujillano Cabello

**Affiliations:** 1 Arnau de Vilanova University Hospital, 25198 Lleida, Spain; 2 IRB-Lleida, Institut de Recerca Biomédica, 25198 Lleida, Spain

**Keywords:** drug resistance, critical care, risk factors, machine learning

## Abstract

**Objectives:** To establish the risk of being a carrier of multidrug-resistant bacteria (MDR) upon ICU admission, according to the risk factors (RFs) from the Spanish “Resistencia Zero” (RZ) project checklist, using machine learning methodology. **Methods:** A retrospective cohort study, conducted with a consecutive sample of patients admitted to the ICU between 2014 and 2016. The study analyzed the RZ RFs for MDR, as well as other pathological variables and comorbidities. The study group was randomly divided into a development group (70%) and a validation group (30%). Several machine learning models were used: binary logistic regression, CHAID-type decision tree, and the XGBOOST methodology (version 2.1.0) with SHAP analysis. **Results:** Data from 2459 patients were analyzed, of whom 210 (8.2%) were carriers of MDR. The risk grew with the accumulation of RF. Binary logistic regression identified colonization or previous infection by MDR, prior antibiotic treatment, living in a nursing home, recent hospitalization, and renal failure as the most influential factors. The CHAID tree detected MDR in 56% of patients with previous colonization or infection, a figure that increased to almost 74% if they had also received antibiotic therapy. The XGBOOST model determined that variables related to antibiotic treatment were the most important. **Conclusions:** The RZ RFs have limitations in predicting MDR upon ICU admission, and machine learning models offer certain advantages. Not all RFs have the same importance, but their accumulation increases the risk. There is a group of patients with no identifiable RFs, which complicates decisions on preventive isolation.

## 1. Introduction

Multidrug-resistant bacteria (MDR) are an escalating concern and are considered a priority in public health. They have been linked to severe infections with poor therapeutic outcomes, increased hospital stays, and higher healthcare costs [1,2,3,4,5,6]. Intensive care units (ICUs) have been identified as major contributors to the emergence of MDR within hospitals, making them key targets for controlling these pathogens [7]. However, epidemiological surveillance studies have shown that a considerable proportion of patients are already carriers of MDR upon ICU admission, either as an infection or colonization [8,9,10].

In response, the Spanish Society of Intensive and Critical Care Medicine and Coronary Units (SEMICYUC), supported by the Ministry of Health, launched the “Resistencia Zero” (RZ) project in 2014. This initiative aimed to reduce the incidence of ICU-acquired infections caused by MDR pathogens by 20%. Secondary objectives included understanding the epidemiology of MDR infections in Spanish ICUs, distinguishing between imported and ICU-acquired MDR cases, promoting and strengthening safety policies within ICUs, and implementing evidence-based safe practices [11,12].

RZ project recommendations include active screening for MDR in all patients upon ICU admission and at least weekly thereafter. Contact precaution measures—hand hygiene, disposable gloves and gowns, and, if possible, single-patient rooms—should be applied to patients at high risk of carrying MDR according to a risk factor (RF) checklist. This checklist includes six recognized RFs: recent hospital admission and antibiotic therapy, prior MDR carriage, renal replacement therapy, and certain chronic conditions that increase colonization risk, such as chronic ulcers and cystic fibrosis.

However, as demonstrated in some studies [8,9], the sensitivity and specificity of these RFs, recognized in the literature and the RZ checklist, are insufficient to efficiently prevent MDR transmission and guide empirical antibiotic therapy.

The application of machine learning (ML) techniques, using variables present at ICU admission, may provide better risk stratification for MDR carriage. Among ML techniques, we aim to use those with clinical interpretability, an appealing feature for their implementation in clinical settings [13].

The goal of this study is to determine the risk of MDR carriage at ICU admission based on the RF checklist from the RZ project, using machine learning methodology.

## 2. Results

**Figure 1:**
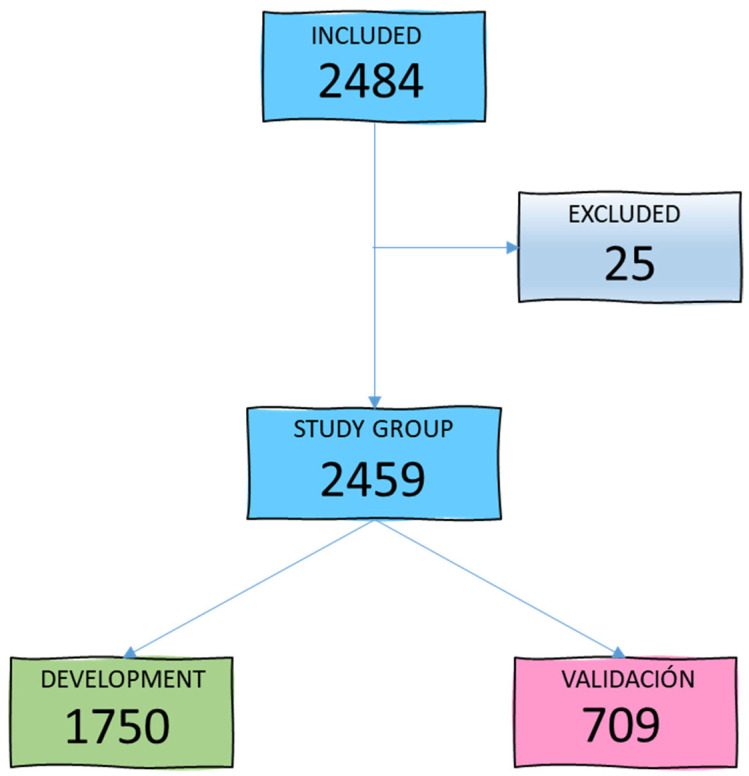
Flowchart of study group selection.

During the study period, 2484 patients aged between 15 and 98 years (mean age 59.4 years) were admitted to our ICU. A total of 25 patients were excluded due to loss of follow-up or lack of microbiological cultures within the first 48 h of admission, leaving 2459 patients for the study group. Figure 1 illustrates the patient selection diagram for the development (GD) and validation (GV) groups.

Of the patients, 62.9% (1547) were male. Among all admissions, 803 (32.7%) met one or more criteria from the RZ checklist, leading to the application of contact precautions for these patients. Table 1 describes the demographic characteristics of the GD and GV groups. Approximately one-quarter of ICU admissions presented at least one risk factor for MDR colonization, with prior hospitalization being the most frequent RF, followed by prior antibiotic use.

A total of 210 patients (8.2%) were identified as MDR carriers, with 222 MDRs isolated (some patients carried more than one MDR). Among these, 92 patients (43.8%) carried extended-spectrum beta-lactamase-producing Enterobacterales (ESBL), 78 (37.1%) carried methicillin-resistant *Staphylococcus aureus* (MRSA), 24 (11.4%) carried MDR *Pseudomonas aeruginosa*, 13 (6.2%) carried *Acinetobacter baumannii*, 5 (2.4%) carried carbapenemase-producing bacteria, and 10 (4.8%) carried other MDR Gram-negative bacteria. In 57 cases (27.1%), MDR presence was associated with infection; in the remainder, it represented colonization. Table 2 details the characteristics of patients with isolated MDR in the GD.

Of the MDR carriers, 132 patients (62.8%) had risk criteria according to the RZ checklist. Among these, 50 patients (38%) had one RF, 53 (40%) had two, and 29 (22%) had three or more RFs, indicating an accumulation of risk. The presence of more RFs correlated with a higher probability of MDR carriage (*p* < 0.001). In 78 MDR carriers (37%), none of the RFs from the RZ project were identified. Table 3 also provides univariable risk values (odds ratios with 95% confidence intervals) and highlights candidate variables for inclusion in multivariable models.

**Figure 2:**
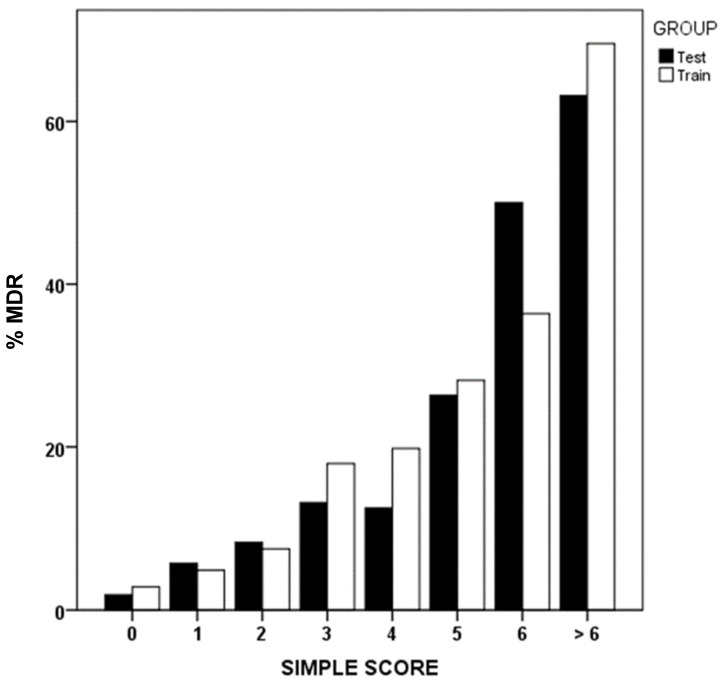
Simple score values and percentage of multidrug-resistant bacteria (MDR). Development (test) and validation (train) groups.

Using binary logistic regression, we identified that prior MDRB colonization or infection, prior antibiotic use, institutionalization, recent hospitalization, and renal failure were the most influential factors associated with MDR presence upon ICU admission (Table 4). Applying the simple score methodology, scores are assigned to each of these variables, reflecting the order of importance and weight of each one as a risk factor. Figure 2 shows MDR prevalence in GD and GV groups, based on the sum of the simple score.

**Figure 3:**
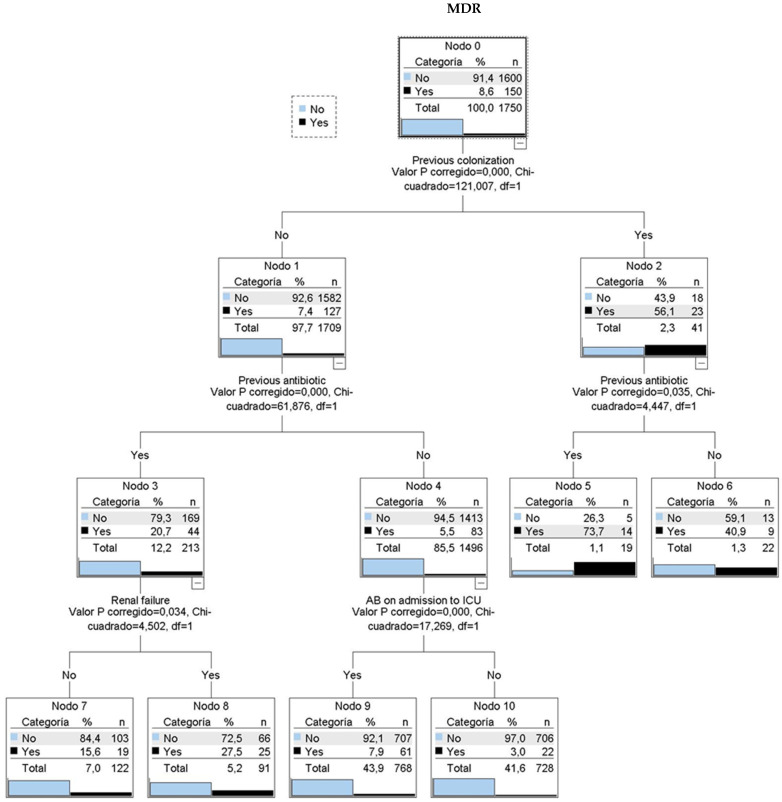
CHAID classification tree. 6 classification rules. MDR: multidrug resistant bacteria. AB: antibiotic treatment. Nodo: node. Categoria: category. Valor P corregido: adjusted *p*-value. Chi-cuadrado: Chi-square.

A CHAID classification tree (Figure 3) identified four key variables: prior colonization, prior antibiotic use, renal failure, and indication for antibiotic therapy at ICU admission. Among patients with prior colonization or infection, 56% had MDR upon ICU admission. This percentage increased to nearly 74% with prior antibiotic use. Patients without prior colonization showed 7.4% MDR prevalence, which rose to 20.7% with prior antibiotic use and 27.5% with additional renal failure history.

**Figure 4:**
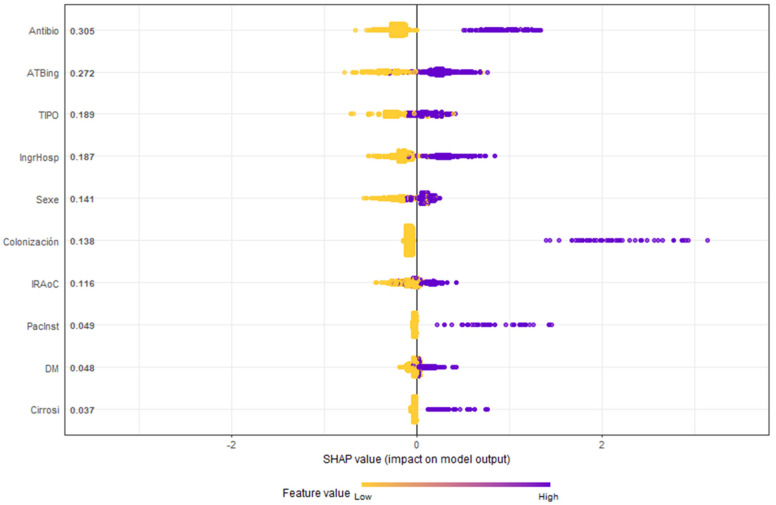
Honeycomb chart shows the evaluation of the variables of the GXBOOST model with SHAP analysis. Antibio: previous antibiotic therapy (7 days in prior month); ATBing: antibiotic therapy upon ICU admission; TIPO: type, urgent medical or surgical diagnosis; IngrHosp: previous hospital admission (5 days in prior 3 months); Sexe: male; Colonización: colonization by MDR; IRAoC: acute or chronic renal failure; PacInst: residence in a nursing home; DM: diabetes mellitus; cirrosi: hepatic cirrhosis.

Figure 4 highlights the SHAP methodology, ranking variables by importance. Notably, antibiotic use before or at ICU admission emerged as the most significant RF.

**Figure 5:**
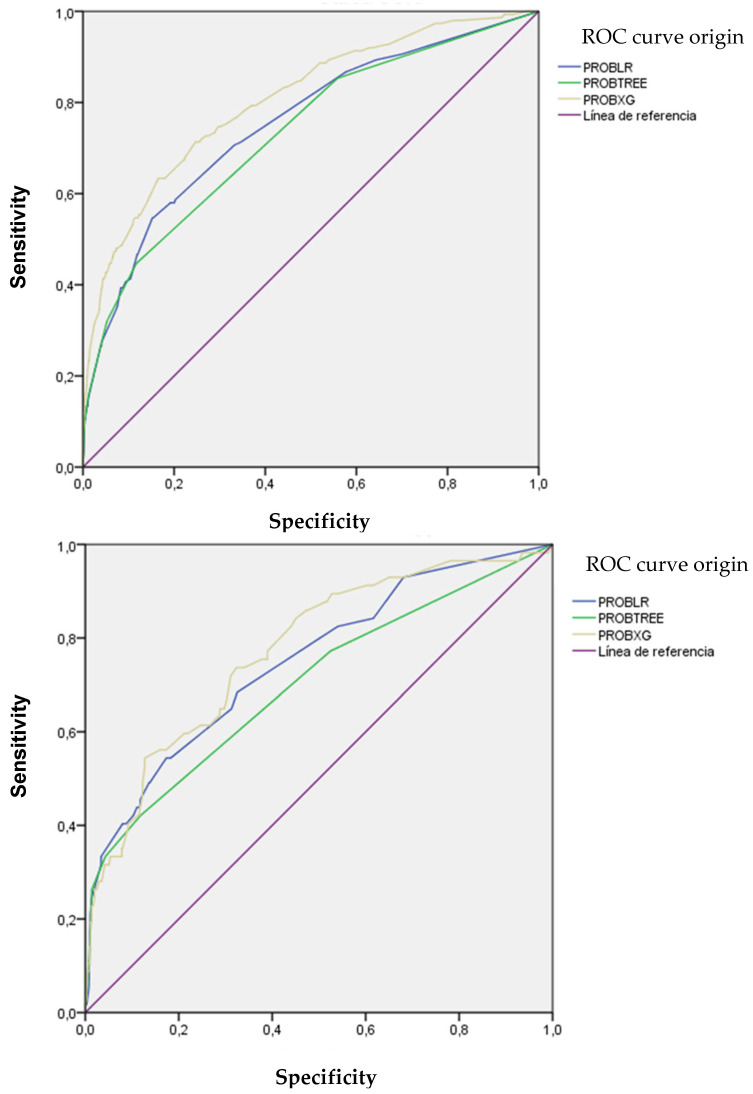
ROC and AUC curves of the models used according to development and validation groups.

**Figure 6:**
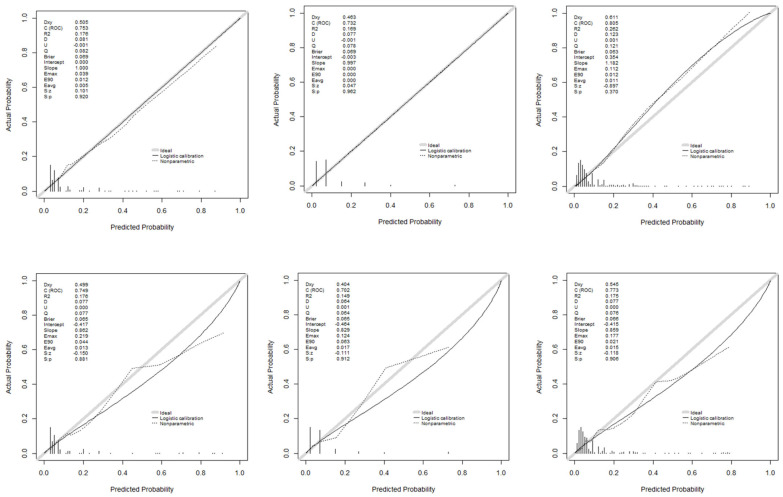
Calibration curves of the models used: development and validation groups.

Figure 5 illustrates the discriminatory capacity of the models in GD and GV, measured by the area under the ROC curve (AUROC). The XGBoost model achieved the highest values, maintaining discriminatory power in GV. Figure 6 presents calibration curves, which show acceptable values, albeit with some loss of calibration in GV.

Applying the RZ criteria, isolating 32.7% of patients effectively captured 62.8% of MDR carriers. A simple score > 1 improved MDR identification to 70.5% while isolating 37.1% of patients. Using the CHAID tree without the antibiotic indication variable, isolating 14.4% of patients achieved 56% MDR identification. Adding this variable increased isolation to 57.3% and detection to 83.1%. This left 1051 patients undetected by these criteria, of whom 35 were MDR carriers. Annex 1 describes these 1051 patients (35 MDR vs. 1016 non-MDR). Undetected MDRs were associated with male gender, unplanned admissions, higher APACHE II scores, and no significant difference in mortality.

## 3. Discussion

The timely identification of MDR upon admission to ICU has clinical and epidemiological implications. Early detection allows for the rapid initiation of appropriate infection control measures, such as selective isolation and contact precautions, which are essential for reducing cross-transmission and subsequent ICU-acquired infections [14,15]. Rapid identification also facilitates timely and appropriate antimicrobial treatment, which is associated with better patient outcomes, including lower mortality and shorter ICU stays [16,17].

When a patient is admitted to the ICU, we can add to the problems caused by their illness if we isolate them because they may be a carrier of MDR. While it is true that isolation and other contact precautions reduce the transmission of MDR, protecting healthcare workers and other patients and preventing outbreaks [18], it also has undesirable effects, such as negative psychological impact, impaired care, adverse reactions, and increased costs related to human, material, and logistical resources [19,20,21,22,23,24]. Patients, especially if they are conscious, tend to become socially and emotionally isolated and may perceive their admission as more depersonalized. Therefore, isolating all patients upon admission to the ICU is not an optimal strategy, but it is necessary to identify patients at higher risk of being MDR carriers in order to optimize healthcare and improve the risk–benefit ratio of contact precautions [25,26,27,28].

The isolation criteria of the RZ project achieve acceptable but not optimal performance: one-third of patients who were MDR carriers were not isolated. A study in Spain published in 2021 [8] reports the unnecessary isolation of almost 70% of patients with RF according to the RZ project. These authors objectively state that a history of colonization or infection by an MDR was the only RF associated with the presence of an MDR upon admission to the ICU. On the other hand, in the Padilla-Serrano series [10], antibiotic therapy prior to admission to the ICU and admission after surgery were the main RFs for rectal colonization by ESBL-producing Enterobacterales. Therefore, it is necessary to look for other models to identify all (or almost all) patients who will be MDR carriers at the time of admission to the ICU. With our models, we improved the number of identifications, although they would require us to isolate a higher percentage of patients upon admission to the unit.

We have detected a significant number of patients with MDR who did not present specific RFs included in the RZ project checklist. For example, in approximately half of the patients in whom MRSA and *A. baumannii* were detected, we were unable to identify any risk factors. It is necessary to know the specific profile of each ICU, based on the premise that the really important information is the incidence of infections and MDR in a unit at a specific time [25]. The ENVIN-HELICS program, with a computerized database for nosocomial infection surveillance applicable to Spanish ICUs, achieves this objective [29,30,31,32].

Not all MDR species have the same RFs for colonizing or infecting a patient. We have also found that not all RFs are equally important, as described in some other studies [10,25,33,34,35]. In addition, our results show that it is easier to assign a higher probability of being an MDR carrier when there is a history of previous colonization, in patients with prolonged hospital stays, or recent use of antibiotic therapy. Our simple score model finds RFs that are more important, such as a history of being an MDR carrier, and that the accumulation of risk factors increases the probability of being an MDR carrier upon admission to the ICU.

The decision rules provided by the CHAID tree model are easy to interpret and help us to identify groups of patients in whom preventive isolation is effective or subgroups of patients who are more problematic in terms of identifying risk factors.

A more complex model, such as XGBOOST, finds better discrimination but becomes a black box when we want to interpret how it classifies risk groups. The SHAP methodology helps us to give importance to variables and provides some explanation of the model. The RF hierarchy it shows helps us to give importance to the use of antibiotics, both before and upon admission to the ICU, as an RF for identifying groups of patients with a high probability of being MDR carriers.

In summary, in our study, we propose machine learning techniques to improve existing predictive models for early detection of MDR upon admission to the ICU. In our series, if we use the RZ risk factor criteria, isolating 32.7% of patients results in effective isolation in 62.8% of MDR carriers. If we use the criterion of a value greater than 1 in the simple score, isolating 37.1% of patients results in an MDR yield of 70.5%. If we use the CHAID tree without the variable “antibiotic treatment” on admission to the ICU, isolating 14.4% of patients gives us a BMR yield of 56%. If we add that variable, we isolate 57.3% of patients to obtain a BMR yield of 83.1%.

Our work has some limitations, such as having been carried out in a single center and the limited sample size, although it was greater than 2000 patients. Other machine learning models could also have been used, but we chose those with the ability to interpret or explain the importance of the RFs found. Our results will need to be validated in other units.

## 4. Materials and Methods

This was a retrospective, observational study conducted at a single center, the Intensive Care Unit of Arnau de Vilanova University Hospital in Lleida (HUAV), a 22-bed multidisciplinary ICU. Data were collected from April 2014 to December 2016, during the implementation of the RZ program in Spain.

Patients included were those admitted to the ICU who underwent active MDR screening through mucosal swabs (nasal, pharyngeal, axillary, rectal) within the first 48 h, as per RZ recommendations, in addition to diagnostic cultures from clinical samples (blood, urine, sputum, tracheal or bronchoalveolar aspirates, surgical wound swabs, or others) based on medical criteria. Patients under 15 years old and those without microbiological cultures performed were excluded.

Patients and/or their families were informed about the microbiological procedures and preventive isolation policy. The HUAV ethics committee (CEIC-3025) approved the study. The development of the models followed the recommendations from the Transparent Reporting of a multivariable prediction model for Individual Prognosis or Diagnosis (TRIPOD) initiative [36].

The study group was randomly divided into a development group (DG) and validation group (VG) (70:30). A patient was considered an MDR carrier at admission if any of the surveillance cultures or clinical samples collected within the first 48 h tested positive for MDR. MDR bacteria included methicillin-resistant *Staphylococcus aureus* (MRSA), vancomycin-resistant *Enterococcus* spp. (VRE), extended-spectrum beta-lactamase-producing Enterobacterales (ESBL), carbapenemase-producing gram-negative bacteria, multidrug-resistant *Pseudomonas aeruginosa* (resistant to more than three common antibiotic families), and carbapenem-resistant *Acinetobacter baumannii*. Other gram-negative bacteria resistant to three or more antibiotic families or producing other resistance mechanisms, such as AMP-C or *Stenotrophomonas maltophilia*, were classified as “others” [11,12].

Variables collected at ICU admission included patient data from the ENVIN-HELICS registry [29] (available at http://hws.vhebron.net/envin-helics/, accessed on 31 March 2024): age, sex, diabetes mellitus (DM), acute or chronic renal failure, immunosuppression, previous malignancy, liver cirrhosis, chronic obstructive pulmonary disease (COPD), malnutrition, and organ transplant. Other data included the source of admission (community, nursing home, other institution, hospital ward, or another ICU), reason for admission (medical, elective surgery, emergency surgery, trauma, or coronary), and whether antibiotic treatment was indicated at ICU admission.

The RZ RFs included hospitalization for more than five days in the last three months, institutionalization, history of MDR carriage, antibiotic therapy for more than seven days in the month before admission, hemodialysis or peritoneal dialysis, and chronic conditions with a high incidence of colonization/infection by MDR (cystic fibrosis, bronchiectasis, chronic ulcers, etc.) [11,12].

Models were developed in the DG and validated in the VG.

Binary logistic regression was used for variable selection, including those with a univariable *p*-value < 0.1 in the multivariable model. A stepwise approach selected significant variables, and coefficients were rounded to the nearest integer for a scoring system (simple score).

A decision tree model using CHAID (chi-square automatic interaction detection) employed cross-validation (five partitions) and a stopping rule with a minimum terminal node size of 10 records.

An XGBoost model was also developed using gradient-boosted classification trees to improve total error. Parameters included a maximum tree depth of 4 and a learning rate of 0.05. The SHAP (SHapley Additive exPlanations) analysis was used to interpret variable importance in the XGBoost model [37].

The models’ accuracy was assessed in both DG and VG by calculating sensitivity (S), specificity (E), positive predictive value (PPV), negative predictive value (NPV), and correct classification percentage. Discrimination was assessed using ROC curves and the area under the curve (AUC), while calibration was evaluated with calibration curves.

Statistical analysis described categorical variables as percentages and continuous variables as medians (interquartile range) due to non-normal distribution (Kolmogorov–Smirnov test). The Mann–Whitney test was used for continuous variables, and the chi-square test for categorical variables, with *p* < 0.05 considered significant. Analyses were performed using SPSS software (version 29.0) and R statistics 4.0.3 with the lrm and SHAPforxgboost packages (version 0.1.3).

## 5. Conclusions

In conclusion, we can say that the risk factors on the checklist that lead to the preventive isolation of patients upon admission to the ICU according to the RZ project have limitations, and the models created with machine learning offer certain advantages. Not all risk factors are equally important, and the decision rules provided by the classification trees identify groups of patients with specific characteristics. The use of antibiotics, both before and upon admission to the ICU, is an RF to be considered. There is a group of patients, whose specific characteristics may vary in each ICU, in whom we did not find any identifiable RFs to consider preventive isolation. And finally, the decision to perform more or fewer isolations, balancing the sensitivity and specificity of the RF and the presented tools, should be made in each unit, depending on the prevalence of MRB, with stricter criteria in settings with high colonization rates.

## Figures and Tables

**Table 1:** Demographic characteristics of ICU patients (*n* = 2459), by Development and Validation groups.

Variable	ALL PATIENTS *n* = 2459	DEVELOPMENT *n* = 1750	VALIDATION *n* = 709	*p*-Value
Age (years)	59.4 ± 16	59.0 ± 16	60.2 ± 16	0.089
Gender (% male)	1547 (62.9)	1092 (62.4)	455 (64.2)	0.409
Medical history				
DM	646 (26.3)	450 (25.7)	196 (27.6)	0.325
COPD	328 (13.3)	229 (13.1)	99 (14.0)	0.562
CKD	729 (29.6)	532 (30.4)	197 (27.8)	0.198
Neutropenia	33 (1.3)	16 (2.3)	17 (1.0)	0.121
Immunosuppression	241 (9.8)	177 (10.1)	64 (9.0)	0.411
Neoplasia	820 (33.3)	572 (32.7)	248 (35.0)	0.275
Cirrhosis	94 (3.8)	73 (4.2)	21 (3.0)	0.157
Solid organ transplant	21 (0.9)	16 (1.0)	17 (2.3)	0.120
Origin				0.833
ED	1864 (75.8)	1332 (76.1)	532 (75.0)	
Nursing home	24 (1.0)	18 (1.0)	6 (0.8)	
Hospital ward	540 (22.0)	377 (21.5)	163 (23.0)	
Other ICU	31 (1.3)	23 (1.3)	8 (1.1)	
Diagnostic				0.763
Medical	1063 (43.2)	764 (43.7)	299 (42.2)	
Elective surgery	800 (32.5)	572 (32.7)	228 (32.2)	
Urgent surgery	261 (10.6)	182 (10.4)	79 (11.1)	
Trauma	335 (13.6)	232 (13.3)	103 (14.5)	
				
TYPE (Medical + urgent surgery)	1324 (53.8)	946 (54.1)	378 (53.3)	0.738
				
Any RZ criterion	803 (32.7)	575 (32.9)	228 (32.2)	0.738
RZ isolation criterion				
Prior hospital admission	635 (25.8)	460 (26.3)	175 (24.7)	0.411
Institutionalized	51 (2.1)	37 (2.1)	14 (2.0)	0.826
Previous colonization	65 (2.6)	41 (2.3)	24 (3.4)	0.144
Previous ATB treatment	325 (13.2)	232 (13.3)	93 (13.1)	0.926
Dialysis	25 (1.0)	19 (1.1)	6 (0.8)	0.592
Chronic patient	60 (2.4)	40 (2.3)	20 (2.8)	0.436
ATB at ICU ADMISSION	1326 (53.9)	955 (54.6)	371 (52.3)	0.312
APACHE II (score)	12 (7–20)	12 (7–20)	13 (7–19)	0.290
ICU length of stay (days)	3 (2–8)	3 (2–7)	3 (2–9)	0.677
Hospital mortality *n* (%)	191 (7.8%)	135 (7.7%)	56 (7.9%)	0.877

Values are expressed as percentages, mean ± standard deviation, or median (interquartile range). MDR: multidrug-resistant bacteria. DM: diabetes mellitus. COPD: chronic obstructive pulmonary disease. CKD: chronic kidney disease. ED: emergency department. RZ: Resistencia Zero program. ATB: antibiotic therapy. APACHE II: Acute Physiology and Chronic Health Evaluation II. *p*-value: from chi-square test or Mann–Whitney test.

**Table 2:** Demographic characteristics of ICU patients according to the isolated MDR organism (*n* = 210).

Variable	ESBL *n* = 92	MRSA *n* = 78	*P. aeruginosa* *n* = 24	*A. baumannii* *n* = 13	OTHERS *n* = 15
Age (years)	61.7 ± 16	59.6 ± 16	63.9 ± 13	63.6 ± 13	67.0 ± 12
Gender (% male)	53 (57.6)	55 (70.5)	20 (83.3)	11 (84.6)	13 (86.7)
Medical history					
DM	39 (42.4)	30 (38.5)	5 (20.8)	3 (23.1)	3 (20.0)
COPD	22 (23.9)	17 (21.8)	6 (25.0)	0	3 (20.0)
CKD	40 (43.5)	35 (44.9)	13 (54.2)	6 (46.2)	10 (66.7)
Neutropenia	0	1 (1.3)	3 (12.5)	0	0
Inmunosuppression	13 (14.1)	3 (3.8)	10 (41.7)	2 (15.4)	3 (20.0)
Neoplasia	31 (33.7)	16 (20.5)	11 (45.8)	3 (23.1)	5 (33.3)
Cirrhosis	8 (8.7)	3 (3.8)	2 (8.3)	2 (15.4)	2 (13.3)
Solid organ transplant	0	2 (2.6)	2 (8.3)	3 (23.1)	1 86.7)
Origin					
ED	52 (56.5)	50 (64.1)	9 (37.5)	8 (61.5)	5 (33.3)
Nursing home	2 (2.2)	0	2 (8.3)	0	0
Hospital ward	34 (37.0)	26 (33.3)	13 (54.2)	5 (38.5)	10 (66.7)
Other ICU	4 (4.3)	2 (2.6)	0	0	0
Diagnostic					
Medical	63 (68.5)	45 (57.7)	15 (62.5)	6 (46.2)	8 (53.3)
Elective surgery	14 (15.2)	18 (23.1)	4 (16.7)	1 (7.7)	0
Urgent surgery	10 (10.9)	8 (10.3)	4 (16.7)	3 (23.1)	6 (40.0)
Trauma	5 (5.4)	7 (9.0)	1 (4.2)	3 (23.1)	1 (6.7)
					
TYPE (Medical + urgent surgery)	73 (79.3)	53 (67.9)	19 (79.2)	9 (69.2)	14 (93.3)
					
Any RZ criterion	61 (66.3)	40 (51.3)	23 (95.8)	6 (46.2)	13 (86.7)
RZ isolation criterion					
Prior hospital admission	45 (48.9)	30 (38.5)	18 (75.0)	6 (46.2)	13 (86.7)
Institucionalized	8 (8.7)	5 (6.4)	2 (8.3)	0	0
Previous colonization	18 (19.6)	17 (21.8)	8 (33.3)	0	1 (6.7)
Previous ATB treatment	40 (43.5)	20 (25.6)	14 (58.3)	4 (30.8)	8 (53.3)
Dyalisis	2 (2.2)	4 (5.1)	0	1 (7.7)	0
Chronic patient	4 (4.3)	4 (5.1)	2 (8.3)	0	0
ATB at ICU ADMISSION	67 (72.8)	52 (66.7)	20 (83.3)	11 (84.6)	14 (93.3)
APACHE II (score)	17 (10–26)	16 (11–23)	19 (14–23)	21 (10–27)	23 (14–30)
Hospital mortality *n* (%)	9 (9.8)	9 (11.5)	3 (12.5)	2 (15.4)	3 (20.0)

Values are expressed as percentages, mean ± standard deviation, or median (interquartile range). ESBL: extended-spectrum beta-lactamase. MRSA: methicillin-resistant *Staphylococcus aureus*. COPD: chronic obstructive pulmonary disease. CKD: chronic kidney disease. ED: emergency department. RZ: Zero Resistance program. ATB: antibiotic therapy. APACHE II: Acute Physiology and Chronic Health Evaluation II.

**Table 3:** Demographic characteristics of the development group (n = 1750), according to detection of MDR carriers.

Variable	NO MRD *n* = 1600	MRD *n* = 150	*p*-Value	OR (CI 95%)	
Age (years)	58.9 ± 16	60.2 ± 17	0.270	1.00 (0.90–1.01)	
Gender (% male)	985 (61.6)	107 (71.3)	0.018	1.55 (1.07–2.24)	C
Medical history					
DM	398 (24.9)	52 (34.7)	0.009	1.60 (1.12–2.28)	C
COPD	199 (12.4)	30 (20.0)	0.009	1.76 (1.15–2.70)	C
CKD	462 (28.9)	70 (46.7)	<0.001	2.15 (1.53–3.02)	C
Neutropenia	15 (0.9)	2 (1.3)	0.636	1.42 (0.32–6.30)	
Inmunosuppression	154 (9.6)	23 (15.3)	0.027	1.70 (1.05–2.73)	C
Neoplasia	528 (33.0)	44 (29.3)	0.360	0.84 (0.58–1.22)	
Cirrhosis	60 (3.8)	13 (8.7)	0.004	2.44 (1.30–4.55)	C
Solid organ tarnsplant	11 (0.7)	6 (4.0)	<0.001	6.01 (2.19–16.5)	C
Origin			<0.001		C
ED	1246 (77.9)	86 (57.3)			
Nursing home	15 (0.9)	3 (2.0)			
Hospital ward	322 (20.1)	55 (36.7)			
Other ICU	17 (1.1)	6 (4.0)			
Diagnostic			<0.001		
Medical	674 (42.1)	90 (60.0)			
Elective surgery	550 (34.4)	22 (14.7)			
Urgent surgery	160 (10.0)	22 (14.7)			
Trauma	216 (13.5)	16 (10.7)			
					
TYPE (Medical + Urgent surgery)	834 (52.1)	112 (74.7)	<0.001	2.70 (1.85–3.96)	C
					
Any RZ criterion	481 (30.1)	94 (62.7)	<0.001	3.90 (2.76–5.53)	
RZ isolation criterion					
Prior hospital admission	384 (24.0)	76 (50.7)	<0.001	3.25 (2.31–4.57)	C
Institucionalized	28 (1.8)	9 (6.0)	<0.001	3.58 (1.66–7.74)	C
Previous colonization	18 (1.1)	23 (15.3)	<0.001	15.9 (8.37–30.2)	C
Previous ATB treatment	174 (10.9)	58 (38.7)	<0.001	5.17 (3.59–7.44)	C
Dialysis	16 (1.0)	3 (2.0)	0.258	2.02 (0.58–7.01)	
Chronic patient	35 (2.2)	5 (3.3)	0.369	1.54 (0.59–3.58)	
ATB at ICU ADMISSION	845 (52.8)	110 (73.3)	<0.001	2.46 (1.69–3.58)	C
APACHE II (score)	12 (7–19)	16 (11–25)	<0.001		
ICU length of stay (days)	3 (2–7)	5 (2–9)	0.001		
Hospital mortality *n* (%)	119 (7.4)	16 (10.7)	0.156		

Values expressed as percentages, mean ± standard deviation, or median (interquartile range). MDR: multidrug-resistant bacteria. DM: diabetes mellitus. COPD: chronic obstructive pulmonary disease. CKD: chronic kidney disease. ED: emergency department. RZ: Zero Resistance program. ATB: antibiotic therapy. APACHE II: Acute Physiology and Chronic Health Evaluation II. *p*-value: using chi-square test or Mann–Whitney test. OR: odds ratio. (CI): confidence interval. (C): candidate variables.

**Table 4:** Logistic regression model of factors influencing the presence of MDR bacteria.

Variable	β Coefficient	OR (CI 95 %)	*p*-Value	Score
Previous colonization	2.267	9.7 (4.8–19.3)	<0.001	4
Previous ATB treatment	1.034	2.8 (1.8–4.4)	<0.001	2
Institucionalizad patient	1.014	2.8 (1.2–6.5)	0.019	2
ATB at ICU admission	0.575	1.8 (1.2–2.7)	0.006	1
Prior hospital admission	0.519	1.7 (1.1–2.5)	0.016	1
Renal failure	0.427	1.5 (1.1–2.2)	0.025	1

MDR: multidrug resistant bacteria. ATB: antibiotics. OR: odds ratio. CI: confidence interval. *Score*: calculated based on the β coefficient value.

## Data Availability

The data presented in this study are available on request from the corresponding author due to privacy and ethical reasons.

## Abbreviations

The following abbreviations are used in this manuscript:

ATB	antibiotic
MDR	multidrug resistant bacteria
ICU	intensive care unit
RZ	Resistencia Zero Project
RF	risk factors
ESBL	extended-spectrum beta-lactamase-producing Enterobacterales
MRSA	methicillin-resistant *Staphylococcus aureus*
SEMICYUC	Sociedad Española de Medicina Intensiva y Unidades Coronarias
ML	machine learning
HUAV	Arnau de Vilanova University Hospital
DG	development group
VG	validation group
VRE	vancomycin-resistant *Enterococcus* spp.
DM	diabetes mellitus
COPD	chronic obstructive pulmonary disease
CKD	chronic kidney disease
ED	emergency department
LR	logistic regression
CHAID	Chi-square automatic interaction detection
SHAP	SHapley Additive exPlanations
S	sensitivity
E	specificity
PPV	positive predictive value
NPV	negative predictive value
AUC	area under the curve
